# Minor Phytocannabinoids as Candidate Multi-Target Modulators of Endoplasmic Reticulum Stress-Linked Proteostasis Failure in Alzheimer’s Disease: A Mechanistic Review

**DOI:** 10.3390/biom16101383

**Published:** 2026-09-22

**Authors:** Hyewon Lee, Sang-Hyuck Park, Han-Jung Chae

**Affiliations:** 1 School of Pharmacy, Jeonbuk National University, 20 Geonji-ro, Deokjin-gu, Jeonju 54907, Republic of Korea; lavine-woni@daum.net; 2 Institute of Cannabis Research, Colorado State University Pueblo, 2200 Bonforte Blvd., Pueblo, CO 81001, USA; sanghyuck.park@csupueblo.edu; 3 Non-Clinical Evaluation Center (NCEC) Biomedical Research Institute, Jeonbuk National University Hospital, 20 Geonji-ro, Deokjin-gu, Jeonju 54907, Republic of Korea

**Keywords:** minor phytocannabinoids, endocannabinoids, cannabis, Alzheimer’s disease, endoplasmic reticulum stress, unfolded protein response, proteostasis

## Abstract

Alzheimer’s disease (AD) involves pathological processes that may limit single-target therapeutic strategies. Endoplasmic reticulum (ER) stress and unfolded protein response (UPR) dysregulation may contribute to proteostasis failure. This review examines whether minor phytocannabinoids from *Cannabis sativa* L.—cannabigerol (CBG), cannabichromene (CBC), cannabinol (CBN), cannabidivarin (CBDV), and tetrahydrocannabivarin (THCV)—warrant investigation as modulators of ER stress-linked proteostasis failure in AD. Evidence is organized around calcium dyshomeostasis, mitochondrial dysfunction and oxidative stress, neuroinflammation, and autophagic impairment as processes that may converge on ER stress, and limited direct evidence for UPR marker changes is evaluated separately. These compounds show overlapping but distinct target profiles involving cannabinoid receptors, transient receptor potential (TRP) channels, peroxisome proliferator-activated receptor γ, mitochondrial and redox pathways, and inflammatory signaling. Available evidence associates CBN primarily with mitochondrial stress pathways, while its involvement in ER–mitochondria signaling remains hypothetical. CBG has been associated with oxidative and inflammatory pathways, THCV with inflammatory signaling, and CBC and CBDV with TRP-related calcium signaling and autophagic pathways. CBDV and CBG have been associated with UPR marker changes in non-neuronal cells, although their functional direction and relevance to AD remain uncertain. Minor phytocannabinoids may serve as pharmacological probes for ER stress-linked proteostasis in AD, and validation in AD-relevant neuronal, organoid, in vivo, and pharmacokinetic studies is required before therapeutic potential can be established.

## 1. Introduction

Alzheimer’s disease (AD) is a complex neurodegenerative disorder in which amyloid-β (Aβ) deposition, abnormal tau phosphorylation, neuroinflammation, synaptic impairment, and progressive neuronal loss are major pathological features [1]. These pathological processes contribute to irreversible cognitive impairments [2,3]. To date, AD treatment has largely relied on symptomatic agents, including N-methyl-D-aspartate (NMDA) receptor antagonists and cholinesterase inhibitors, with the more recent addition of Aβ-directed antibodies, such as lecanemab and donanemab, which have introduced a disease-modifying treatment strategy for early AD. However, the clinical benefits of these antibodies remain limited, especially after substantial neuronal and synaptic injury has occurred [4,5,6,7]. The incomplete efficacy of these largely single-target approaches reflects the pathobiological complexity of AD, in which Aβ and tau pathologies are intertwined with broader disturbances in protein folding, maturation, and clearance systems [8,9]. Thus, AD may be viewed not only as a disorder of Aβ and tau accumulation but also as a chronic proteostasis disorder in which various protein quality control systems progressively fail [8,9,10,11].

Endoplasmic reticulum (ER) stress links several AD-related abnormalities to proteostasis failure. The ER is a central folding and quality control machinery for a diverse repertoire of secretory, membrane-associated, receptor, signaling, and metabolic proteins, many of which are required for neuronal homeostasis [12,13]. When ER stress is acute or reversible, the unfolded protein response (UPR) serves as a protective, adaptive response that restrains the entry of nascent proteins into the ER, induces molecular chaperones to expand folding capacity, and promotes the clearance of misfolded substrates via ER-associated degradation [12]. However, in chronic neurodegenerative conditions such as AD, persistent ER stress may shift UPR signaling from adaptive compensation to insufficient proteostatic control [9,10,11]. This chronic failure is relevant to AD because Aβ and tau pathology, mitochondrial dysfunction, Ca^2+^ dyshomeostasis, neuroinflammation, and autophagic impairment can reinforce the ER proteotoxic burden [9,10,14,15,16,17]. Thus, ER stress in AD should not be viewed solely in terms of canonical UPR sensor activation involving protein kinase R-like ER kinase (PERK), inositol-requiring enzyme 1α (IRE1α), and activating transcription factor 6 (ATF6). Rather, ER stress reflects a broader deterioration of protein maturation and quality control systems that regulate multiple disease-associated protein networks [9,10,11,12]. Figure 1 summarizes this conceptual shift by contrasting adaptive UPR signaling, which maintains proteostasis, with maladaptive UPR signaling associated with chronic ER stress, proteostasis failure, and neuronal dysfunction.

The endocannabinoid system (ECS) is altered in AD-affected brain regions [18,19,20]. In parallel, ECS-related signaling regulates several processes relevant to ER stress-linked proteostasis, including synaptic transmission, neuroinflammation, Ca^2+^ homeostasis, mitochondrial function, oxidative stress, autophagy, and cell survival [21,22,23]. Thus, cannabinoid-related signaling can be considered in relation to ER stress-linked proteostasis failure, rather than only as an isolated receptor system. To date, AD-related cannabinoid studies have mainly examined cannabidiol (CBD) and Δ^9^-tetrahydrocannabinol (THC), whereas the pharmacological relevance of minor phytocannabinoids has received comparatively less attention [19,20,24]. This disparity is notable because *Cannabis sativa* L. contains more than 150 phytocannabinoids, the majority of which remain pharmacologically uncharacterized [24,25]. Cannabigerol (CBG), cannabichromene (CBC), cannabinol (CBN), cannabidivarin (CBDV), and tetrahydrocannabivarin (THCV) are representative minor phytocannabinoids with distinct but partially overlapping pharmacological profiles [24,26]. Although typically present at lower concentrations than THC or CBD, these compounds engage overlapping but compound-specific targets and pathways. Their reported targets and pathways include cannabinoid receptor 1 (CB1R), a presynaptic receptor that regulates neuromodulation and synaptic transmission [27]; cannabinoid receptor 2 (CB2R), which is associated with glial and immune regulation and can attenuate inflammatory signaling [19,20,28,29]; transient receptor potential (TRP) channels, which participate in sensory transduction, Ca^2+^-dependent signaling, and cellular stress responses [22,23,26]; peroxisome proliferator-activated receptor γ (PPARγ)-related pathways, which regulate metabolic and anti-inflammatory transcriptional programs [30]; and mitochondrial stress-related pathways, which contribute to redox balance, bioenergetic control, and cellular stress adaptation [23,24,31]. Therefore, minor phytocannabinoids can be considered candidate modulators of ER stress-linked proteostasis in AD.

This review discusses CBG, CBC, CBN, CBDV, and THCV in the context of ER stress-related proteostasis failure in AD. Importantly, direct evidence that any of these five minor phytocannabinoids modulate ER stress in AD-relevant neuronal models is currently lacking, and most mechanistic links discussed in this review are derived from non-neuronal systems or non-AD disease models. These links should therefore be regarded as hypothesis-generating rather than as evidence of established neuroprotective mechanisms in AD. Accordingly, rather than treating these compounds as conventional single-target agents, the discussion is organized around four disease-relevant processes that can intensify chronic ER stress and UPR failure, including Ca^2+^ dyshomeostasis, mitochondrial dysfunction and oxidative stress, neuroinflammation, and autophagic impairment. This framework aligns each compound with the pathological processes supported to varying degrees by the available evidence while distinguishing proposed mechanistic links from experimentally validated effects.

As this article is a narrative mechanistic review rather than a systematic review, relevant literature was identified primarily through PubMed searches using combinations of terms related to Alzheimer’s disease, endoplasmic reticulum stress, unfolded protein response, proteostasis, and the five minor phytocannabinoids discussed in this review. Relevant literature published through August 2026 was considered. Primary experimental studies were prioritized for compound-specific pharmacological and mechanistic claims, whereas review articles were used mainly to provide broader pathway context. No Preferred Reporting Items for Systematic Reviews and Meta-Analyses (PRISMA)-based study-selection procedure or formal risk-of-bias assessment was performed.

## 2. ER Proteostasis Failure and UPR Dysregulation in AD

### 2.1. Proteostasis Failure and Chaperone Dysfunction in AD

The ER is an important site for protein folding, modification, and trafficking and is responsible for the maturation of a substantial portion of the cellular proteome [12,13]. Within the ER lumen, molecular chaperones and foldases help newly synthesized proteins acquire proper three-dimensional structures before proceeding through the secretory pathway [12,32,33]. Glucose-regulated protein 78/binding immunoglobulin protein (GRP78/BiP) serves as a key ER chaperone that maintains UPR sensors in a regulated state and helps reduce the accumulation of misfolded proteins [32,33]. Under basal conditions, GRP78/BiP remains associated with the three main ER stress sensors (IRE1α, PERK, and ATF6), thereby maintaining them in an inactive state [32,33]. Other ER chaperones and foldases, including protein disulfide isomerase (PDI), calnexin, calreticulin, and glucose-regulated protein 94 (GRP94), support disulfide bond formation, glycosylation, and oxidative protein folding [12,33].

In AD, ER proteostasis appears to be disrupted, as reflected by ER stress marker induction, altered UPR signaling, and changes in chaperone- and foldase-mediated protein quality control [14,34]. In postmortem AD brain tissue, GRP78/BiP and PDI levels are elevated, and the phosphorylation of the UPR sensors PERK and IRE1α is also increased [14,32,33,34]. These findings indicate activation of the ER quality control machinery and upstream UPR signaling, consistent with chronic ER stress in AD-affected brain regions. Moreover, the accumulation of misfolded or aggregation-prone Aβ species and hyperphosphorylated tau potentially contributes to the ER proteotoxic burden by disrupting protein-folding homeostasis, ER-associated degradation (ERAD), and UPR signaling [10,11,14,15,34]. Aβ oligomers have been implicated in abnormal ER Ca^2+^ release involving ryanodine receptors (RyRs) and inositol 1,4,5-trisphosphate receptors (IP_3_Rs), thereby disrupting ER Ca^2+^ homeostasis [16,17,35]. Because chaperones such as calnexin and calreticulin require Ca^2+^ for activity, disrupted ER Ca^2+^ homeostasis can impair their function, thereby compromising ER protein-folding capacity [12,16,17].

Several post-translational modifications may further affect ER proteostasis in AD. S-nitrosylation (SNO) of PDI under nitrosative stress can impair its isomerase and chaperone activities, leading to accumulation of polyubiquitinated misfolded proteins and sustained UPR activation [32,36]. Altered communication between the ER and mitochondria may also contribute to AD-related ER stress. These contact regions, referred to as mitochondria-associated membranes (MAMs), coordinate inter-organelle Ca^2+^ signaling and help support mitochondrial energy production required for ER protein quality control [16,17,37]. ER proteostasis in AD may be disrupted by abnormalities in chaperone function and ER stress-related protein regulation [10,32,33]. Altered Ca^2+^ handling and ER–mitochondria communication provide additional sources of proteostatic stress [16,17,37]. These changes may increase neuronal vulnerability to unresolved protein misfolding and chronic ER stress [10,11,14,34]. These converging mechanisms are summarized in Figure 2 as a proposed model of ER proteostasis disruption in AD.

### 2.2. UPR Activation and Maladaptive Signaling in AD

Disruption of ER proteostasis activates UPR signaling through IRE1α, PERK, and ATF6, which function as membrane-bound stress-sensing proteins in the ER [12,38]. Under acute or mild ER stress, UPR acts mainly as an adaptive response by increasing chaperone expression, promoting ERAD, reducing global protein synthesis, and supporting autophagy [11,12,38]. However, in chronic neurodegenerative conditions such as AD, prolonged UPR activation may shift toward pro-apoptotic and inflammatory signaling [10,11,38].

Upon ER stress, the dissociation of GRP78/BiP permits IRE1α molecules to cluster and undergo activating phosphorylation [32,38]. In addition to its canonical role in X-box binding protein 1 (XBP1) splicing, IRE1α can help reduce the protein-folding burden of the ER by initiating regulated IRE1-dependent decay (RIDD), through which selected transcripts encoding ER-targeted or secretory proteins are cleaved [32,38]. When ER stress is sustained, the cytosolic domain of IRE1α can recruit stress-signaling components, including tumor necrosis factor receptor-associated factor 2 (TRAF2) and apoptosis signal-regulating kinase 1 (ASK1), thereby engaging c-Jun N-terminal kinase (JNK)-associated and nuclear factor-kappa B (NF-κB)-associated inflammatory and apoptotic pathways [10,32,38]. Under nitrosative or inflammatory stress conditions relevant to AD, IRE1α can be S-nitrosylated, thereby reducing its endoribonuclease activity, impairing *XBP1* mRNA splicing, and decreasing the generation of spliced XBP1 (XBP1s) [32,33,39,40]. This may weaken adaptive IRE1α–XBP1s signaling while allowing IRE1α-associated JNK stress signaling to persist, thereby shifting UPR responses from proteostatic adaptation to maladaptive stress responses.

Upon activation, PERK phosphorylates eukaryotic initiation factor 2α (eIF2α) on Ser51. This modification temporarily dampens bulk mRNA translation, thereby reducing the flow of nascent polypeptide chains into the ER [12,33,38]. Despite this general translational brake, eIF2α phosphorylation permits preferential translation of activating transcription factor 4 (ATF4). During unresolved or persistent ER stress, ATF4 contributes to the induction of C/EBP homologous protein (CHOP) [12,33,38,41]. CHOP can further enhance oxidative ER–mitochondria stress by inducing ER oxidoreductin 1α (ERO1α), which promotes reactive oxygen species (ROS) generation and stimulates IP_3_R-dependent ER Ca^2+^ release [33,41]. Activation of the PERK–eIF2α–ATF4–CHOP pathway has been reported in AD brain tissue, and similar ER stress marker changes have been observed in AD transgenic models during Aβ accumulation [14,34,42]. Aberrant PERK–eIF2α signaling may contribute to impaired protein synthesis, synaptic plasticity, and memory in AD mouse models [43].

In addition, ER stress induces the Golgi-directed trafficking of ATF6. In the Golgi, sequential cleavage by site-1 protease and site-2 protease releases the N-terminal cytosolic transcription factor domain of ATF6 [12,38,44]. The cleaved fragment then enters the nucleus and promotes the expression of ER chaperones and UPR regulators, including GRP78/BiP, GRP94, and XBP1 [12,44]. ATF6 activation is generally considered protective, but sustained or dysregulated ATF6 signaling may also contribute to maladaptive UPR signaling depending on the disease stage, cell type, and stress severity [33,38].

Overall, UPR signaling in AD should not be interpreted simply as a bystander response. In chronic AD-related stress, persistent activation of upstream UPR sensors may coexist with insufficient adaptive downstream responses, including XBP1s-dependent transcription, chaperone induction, and ERAD [10,11,32,38]. Disruption of XBP1s nuclear translocation, impaired interaction with phosphoinositide 3-kinase (PI3K) regulatory subunits p85α/β, or SNO-mediated suppression of IRE1α ribonuclease activity may weaken the adaptive IRE1α–XBP1s pathway and contribute to unresolved protein misfolding [32,33,39,40]. In addition, altered ER–mitochondria communication at MAMs may impair Ca^2+^ transfer and mitochondrial energy production, thereby limiting energy-dependent protein refolding and degradation processes [16,17,37]. These findings suggest that chronic UPR dysregulation may contribute to proteostasis failure and neuronal vulnerability in AD [10,11]. Together, these observations underscore the need for further evaluation of approaches that restore adaptive UPR function rather than broadly suppressing ER stress responses [11,38].

## 3. Cannabinoid-Sensitive Pathways in Alzheimer’s Disease

The UPR has emerged as a promising intervention point in AD, but selectively targeting its three branches presents substantial challenges since PERK, IRE1α, and ATF6 each perform critical functions under normal cellular conditions [10,11,38]. Therefore, targeting the upstream processes that sustain chronic ER stress may be more appropriate than directly inhibiting or activating UPR sensors. The ECS is relevant to this strategy because ECS-related and phytocannabinoid-sensitive targets influence processes that overlap with ER stress-related pathways, including Ca^2+^ homeostasis, mitochondrial bioenergetics, oxidative stress, neuroinflammation, and autophagic flux [22,23,45]. Also, these processes are major contributors to ER stress and UPR dysregulation in AD [10,46,47,48]. Cannabinoid signaling has also been reported to affect ER homeostasis and UPR-related markers, including PERK, IRE1α, and ATF6, although these effects may differ depending on receptor subtype, cell type, and stress condition [49]. ECS signaling is altered in AD-affected brain regions [18,19,20,21]. This makes ECS-related pharmacology relevant to the upstream processes that shape UPR function in AD.

Structurally, the ECS encompasses CB1R and CB2R as its primary receptors, the endogenous lipid ligands anandamide and 2-arachidonoylglycerol, and a suite of metabolic enzymes responsible for their synthesis and catabolism [22,50]. In the mammalian brain, CB1R is highly enriched at presynaptic sites, where it modulates neurotransmitter release and contributes to synaptic plasticity [27,50]. Within the central nervous system (CNS), CB2R expression is relatively low under basal conditions but is increased in glial and immune-competent cells, most notably microglia and astrocytes, under neuroinflammatory conditions, where it can modulate glial activation and attenuate inflammatory signaling, at least in part, by limiting pro-inflammatory cytokine production [28,29]. Postmortem evidence indicates altered ECS receptor expression in AD, with reduced CB1R levels in AD-vulnerable brain regions and marked CB2R elevation in reactive glial cells associated with Aβ plaque deposition [18,20,28,29].

Further, phytocannabinoids interact with several non-canonical targets outside the classical CB1R/CB2R system. The non-canonical targets include members of the TRP channel family, particularly transient receptor potential vanilloid 1 (TRPV1) and transient receptor potential ankyrin 1 (TRPA1), alongside the nuclear receptors peroxisome proliferator-activated receptor alpha (PPARα) and PPARγ [22,23,26]. TRPV1 channels in hippocampal neurons regulate Ca^2+^-dependent signaling and have been linked to autophagic flux in tau-related models [51]. PPARγ acts as a transcriptional regulator that controls anti-inflammatory and metabolic gene programs in neurons, astrocytes, and microglia [30,52]. These targets regulate Ca^2+^ homeostasis, mitochondrial function, oxidative stress, inflammatory signaling, and autophagy, thereby linking cannabinoid-related pharmacology to ER stress-relevant pathways [10,22,23,46,47,48].

Broader ECS dysregulation and cannabinoid-based therapeutic modulation in AD have also been discussed in preclinical and review studies [19,20,53]. Pharmacological modulation of CB1R, CB2R, TRPV1, and PPARγ has been linked to reduced Aβ-associated neurotoxicity, attenuated neuroinflammation, regulation of tau- or Ca^2+^-related stress signaling, or improved cognitive and neuroprotective outcomes in AD-related or tau-related experimental models [19,20,51,52]. The intersection of ECS-related and phytocannabinoid-sensitive targets with ER stress-relevant processes provides a rationale for investigating minor phytocannabinoids in this context (Figure 3). However, target or pathway overlap alone does not establish that a given compound modulates ER stress in AD. In this review, such overlap is therefore used as a hypothesis-generating framework for identifying testable compound–pathway relationships rather than as evidence of established mechanistic relevance or therapeutic benefit.

## 4. Minor Phytocannabinoids: Pharmacological Overview

Minor phytocannabinoids are structurally diverse compounds derived from *Cannabis sativa* and are typically less abundant than THC or CBD in non-enriched cannabis or hemp materials [54,55,56]. An additional consideration is the composition of the cannabinoid preparation used in pharmacological studies. Some studies cited in this review employed cannabinoid-enriched extracts rather than isolated compounds [57,58]. In such preparations, co-occurring cannabinoids, including THC, CBD, or other minor constituents, may contribute to observed receptor, channel, or in vivo effects. Therefore, effects observed with enriched extracts should not be attributed solely to the nominal minor phytocannabinoid unless preparation composition and purity are adequately characterized and, where possible, supported by isolated-compound controls. Cannabinoid abundance varies substantially according to chemotype, cultivar, plant tissue, developmental stage, cultivation conditions, post-harvest processing, and analytical method. Therefore, the term “minor” should be interpreted as a relative abundance-based designation rather than an indication of limited biological relevance. In THC-dominant cannabis flowers, tetrahydrocannabinolic acid or total THC commonly occurs at percent-level concentrations, often in the range of approximately 10 to more than 20% by dry weight in commercial or medical cannabis datasets [54,55]. In CBD-dominant hemp cultivars, cannabidiolic acid may reach high mg/g dry weight levels, corresponding to a percent-level abundance in selected cultivars [56]. In contrast, individual minor cannabinoids, such as CBG, CBC, CBN, CBDV, and THCV, are usually detected at substantially lower levels in non-enriched plant material, often below 1% by dry weight or below 10 mg/g, although CBG-, cannabigerolic acid-, or varin-enriched chemovars can contain higher amounts [54,55,56]. The chemical structures of CBG, CBC, CBN, CBDV, and THCV are shown in Figure 4 to facilitate comparison of their structural features. As described above, CBG, CBC, CBN, CBDV, and THCV engage partially overlapping but distinct molecular targets and stress-related pathways, including CB1R/CB2R activity, TRP channels, PPARγ-related signaling, and mitochondrial or oxidative stress-related responses [24,26,30,31]. These target and pathway profiles overlap with ER stress-relevant upstream processes in AD, including Ca^2+^ dyshomeostasis, mitochondrial dysfunction and oxidative stress, neuroinflammation, and autophagic impairment. The primary targets, pharmacological properties, and proposed relevance of these minor phytocannabinoids to ER stress are summarized in Table 1. These proposed links should be interpreted as mechanistic hypotheses rather than established AD therapeutic mechanisms.

## 5. Minor Phytocannabinoids as Potential Modulators of ER Stress Pathways

The following sections organize the available evidence around four upstream mechanisms depicted in Figure 5, namely Ca^2+^ dyshomeostasis, mitochondrial dysfunction and oxidative stress, neuroinflammation, and autophagic impairment. These domains were selected because they represent major ER stress-relevant processes in AD [10,11,16,17,46,47,48]. Because minor phytocannabinoids act on targets linked to these ER stress-relevant pathways [22,23], the evidence is organized according to the ER stress-related processes that each compound may influence, rather than by treating these compounds as conventional single-target agents. The limited direct evidence that selected minor phytocannabinoids may modulate UPR-related markers is then considered separately, because these data are not readily assigned to one upstream mechanism.

### 5.1. Ca^2+^ Dyshomeostasis and ER Stress

Intracellular calcium dysregulation is an important contributor to ER stress in AD [10,46]. Because the ER functions as the major intracellular Ca^2+^ reservoir, depletion of ER Ca^2+^ can compromise ER-resident chaperones and Ca^2+^-dependent folding machinery [12,46]. Aβ oligomers may disrupt intracellular Ca^2+^ homeostasis through pore-forming activity and by sensitizing ER Ca^2+^ release pathways, including IP_3_R- and RyR-dependent signaling [10,35,46,79,80,81]. Also, disrupted ER–mitochondria calcium transfer through the MAMs may further amplify this dysfunction [16,17].

Several minor phytocannabinoids have been reported to modulate intracellular calcium signaling through TRP channels and, in some cases, other Ca^2+^-regulatory targets. CBG has been reported to antagonize transient receptor potential melastatin 8 (TRPM8), and this pharmacological activity may allow CBG to reshape TRP-associated Ca^2+^ signaling under conditions of disrupted ER Ca^2+^ regulation [57,59]. The relevance of this TRPM8-related mechanism to ER Ca^2+^ regulation in AD remains indirect and requires validation in neural models. CBC and CBDV may influence TRP-related calcium signaling primarily through TRPA1- and TRPV1-related mechanisms, respectively [57,64,75,82]. Here, TRPA1 has been linked to reactive electrophile sensing and stress-related Ca^2+^ signaling [64,82], whereas TRPV1-mediated Ca^2+^ influx may influence ER Ca^2+^ homeostasis and ER–mitochondria Ca^2+^ coupling under specific cellular conditions [45,71,74]. CBN may influence the ER–mitochondria calcium axis through putative sigma-1 receptor (S1R)/MAM-related mechanisms, although direct CBN–S1R engagement in AD-relevant neuronal models remains to be validated [67,68]. Collectively, these observations indicate that minor phytocannabinoids should not be interpreted as uniformly increasing or reducing Ca^2+^ signaling. Their relevance to ER stress depends on the amplitude, duration, and subcellular location of TRP-linked Ca^2+^ signals. In this context, TRPM8 antagonism, TRPA1/TRPV1 activation, and TRPV1 desensitization may represent distinct forms of Ca^2+^ signal remodeling rather than mutually opposing mechanisms. Despite their different upstream effects, these Ca^2+^ signals may converge functionally on ER Ca^2+^ homeostasis by altering the balance between cytosolic Ca^2+^ signaling, ER Ca^2+^ release, and sarco/endoplasmic reticulum Ca^2+^ ATPase (SERCA)-dependent ER refilling [45,46]. Sustained disruption of this balance may reduce ER luminal Ca^2+^ availability and thereby impair Ca^2+^-dependent ER chaperone and protein-folding functions [12,46]. This convergence does not imply that CBG, CBC, or CBDV directly modulate SERCA, which remains to be experimentally determined. Transient or localized TRP-linked Ca^2+^ signaling may support adaptive autophagy, whereas sustained Ca^2+^ entry or depletion of ER Ca^2+^ stores may increase ER proteostatic burden.

### 5.2. Mitochondrial Stress, Redox Imbalance, and ER Proteostasis

Mitochondrial dysfunction can contribute to ER stress in AD and may act as both an upstream trigger and downstream consequence of UPR dysregulation [10,11]. Impaired electron transport chain activity, including reduced complex I and IV function observed in AD brain tissue, can elevate mitochondrial ROS production, thereby promoting oxidative damage to ER-resident proteins, impairing SERCA pump activity, and contributing to UPR activation [10,11,17,83,84,85,86]. CHOP-mediated apoptotic signaling downstream of PERK may further target mitochondria, creating a feed-forward loop between mitochondrial injury and ER stress [10,11,41]. Therefore, preserving mitochondrial integrity may indirectly reduce the ER proteotoxic burden by limiting ROS production, Ca^2+^ dysregulation, and bioenergetic failure. Recent comparative transcriptomic analyses of the buffalo cerebellum and metabolically active peripheral organs further suggest that the mammalian brain exhibits a distinct mitochondrial regulatory program, with relatively greater emphasis on regulated transport, signaling, detoxification and protein repair, and maintenance of mitochondrial gene expression than on bulk bioenergetic throughput [87,88]. This brain-enriched profile includes pathways involved in mitochondrial transcriptional elongation, RNA surveillance, mitochondrial DNA maintenance, and repair of oxidatively damaged proteins, and several of these regulatory patterns appear to be conserved between buffalo and human brain datasets [87,88]. Such tissue-specific mitochondrial organization provides an additional physiological basis for caution when extrapolating stress responses observed in proliferative peripheral cells to neural systems.

CBN has been reported to limit oxidative stress-induced neuronal cell death through mitochondrial pathway modulation, an effect that is largely independent of cannabinoid receptor activation [31]. These effects may involve the preservation of mitochondrial function and bioenergetic stability. In addition, CBN may influence the mitochondrial–ER stress axis through putative S1R/MAM-related mechanisms, as S1R is a MAM-associated chaperone that regulates ER–mitochondria communication and stress-responsive UPR signaling [67,69]. In a 3-nitropropionate-lesioned Huntington’s disease model, CBG restored antioxidant defense parameters, with effects observed for catalase, superoxide dismutase (SOD), and glutathione (GSH), without activating nuclear factor erythroid 2-related factor 2 (Nrf2)-dependent signaling [61]. Together with the CBN-related mitochondrial findings described above, these observations suggest that selected minor phytocannabinoids, particularly CBN and CBG, may influence ER stress-related processes through mitochondrial and oxidative stress-related pathways.

### 5.3. Neuroinflammation and Inflammatory Amplification of UPR Signaling

Chronic neuroinflammation can amplify ER stress through two interrelated mechanisms. First, microglial pro-inflammatory mediators, notably tumor necrosis factor alpha (TNF-α) and interleukin-1 beta (IL-1β), can activate NF-κB-centered inflammatory signaling and stress kinase cascades that intersect with IRE1α- and PERK-associated UPR pathways, thereby favoring maladaptive and pro-apoptotic stress responses [9,10,47]. Second, sustained JNK activation downstream of IRE1α may amplify inflammatory gene expression, creating a feed-forward loop between UPR signaling and neuroinflammation [9,10,47]. Therefore, reducing neuroinflammatory stimuli may help limit the inflammatory amplification of ER stress in AD [9,11].

Minor phytocannabinoids may influence inflammatory pathways through distinct but potentially complementary mechanisms. In preclinical neurodegeneration models, THCV has been associated with reduced inflammatory responses and neuroprotective effects [76]. CBG may engage anti-inflammatory signaling pathways, including PPARγ-related mechanisms, based mainly on preclinical inflammatory and neuroinflammatory models [30,60]. CBC has been reported to reduce macrophage inflammatory mediators, potentially through TRP channel modulation and inhibition of prostaglandin E2 synthesis, and may also influence inflammatory signaling through relatively low-potency CB2R agonist activity [64,65]. CBDV has also been linked to reduced NF-κB-associated inflammatory gene expression in neuroinflammation models [73]. These anti-inflammatory effects raise the possibility that minor phytocannabinoids indirectly attenuate inflammatory amplification of IRE1α–JNK and PERK–CHOP signaling in AD, but their direct effects on these pathways in AD models remain to be defined [9,10,47].

### 5.4. Autophagic Impairment and Proteostasis

Autophagy is a major mechanism for clearing misfolded proteins, damaged organelles, and proteasome-resistant aggregates that accumulate during ER stress [11,48]. In AD, autophagic flux is impaired at multiple steps, including autophagosome formation, lysosomal acidification, and cathepsin-mediated degradation, leading to the accumulation of autophagic vacuoles containing Aβ and hyperphosphorylated tau [48,51,89]. This autophagic failure may exacerbate the ER proteotoxic burden by reducing ERAD substrate clearance and increasing the load of misfolded proteins that must be processed by the UPR [11,15]. Thus, promoting autophagic flux may help alleviate ER stress-linked proteostasis failure in AD.

In tau-related models, pharmacological activation of TRPV1 has been linked to enhanced autophagic flux and improved lysosomal function [51]. CBDV can activate TRPV1 [26,75], whereas CBC shows low-efficacy TRPV1 activity in cannabinoid profiling studies, in addition to its stronger TRPA1-related activity [57]. TRPV1-linked autophagy has been associated with mechanistic target of rapamycin (mTOR) suppression and lysosomal activation, a mechanism that may enhance the clearance of misfolded proteins [51]. However, these observations do not establish that TRPV1 activation by CBDV or CBC is uniformly beneficial. As discussed in Section 5.1, the consequences of TRP-mediated Ca^2+^ signaling depend on signal amplitude, duration, and subcellular localization, and sustained Ca^2+^ entry may instead increase ER proteostatic stress [45,46]. Thus, whether CBDV- or CBC-associated TRPV1 signaling supports or impairs autophagic proteostasis is likely to be context-dependent and requires direct evaluation in AD-relevant neuronal models.

### 5.5. Potential Modulation of UPR Signaling

The direct evidence for CBDV- and CBG-associated UPR marker modulation currently derives from a single study using HTR-8/SVneo trophoblast cells [71]. In that study, CBDV and CBG were examined at 1–10 μM. At 5 μM, both compounds increased heat shock protein family A member 5 (*HSPA5*, encoding GRP78/BiP) and *XBP1s* transcript levels, whereas CBDV additionally increased *ATF4* transcript levels together with phosphorylated eIF2α (p-eIF2α) and CHOP protein levels and initiator caspase-8/-9 activity. Neither compound activated effector caspases-3/-7 or induced poly(ADP-ribose) polymerase-1 (PARP-1) cleavage [71].

These findings should therefore be interpreted as evidence of context-dependent UPR marker responsiveness rather than beneficial ER stress modulation. Because the CBDV- and CBG-associated responses were observed under specific experimental conditions, their functional significance may vary with dose and cellular context [71]. More broadly, whether UPR signaling is adaptive or maladaptive depends on stress intensity and duration, cell type, and disease stage [12,38,90]. Changes in individual UPR markers alone are insufficient to distinguish these outcomes. Transient induction of adaptive chaperone responses, including GRP78/BiP, together with recovery of proteostasis, may be consistent with an adaptive response, whereas sustained PERK–eIF2α–ATF4–CHOP signaling accompanied by apoptotic or functional deterioration would support maladaptive stress activation [12,38,41,90].

However, these findings were obtained in proliferative placental trophoblast cells and should not be directly extrapolated to post-mitotic neurons. Trophoblast cells and neurons differ in receptor context, TRPV1 expression, Ca^2+^ handling, and basal proteostatic demands. Accordingly, validation in AD-relevant neuronal models is required before these effects can be assigned neurobiological or translational significance. CBDV is emphasized within the present framework not because it has stronger direct evidence for UPR modulation than CBG, but because its TRPV1-related Ca^2+^ signaling [26,75] provides a mechanistic rationale for linking CBDV to ER stress-related calcium homeostasis [45,71,74] and TRPV1-associated autophagic proteostasis [51]. This emphasis does not imply that CBG should be deprioritized; both compounds require parallel validation in AD-relevant neuronal models.

The S1R at MAMs represents a separate candidate pathway through which minor phytocannabinoids may influence UPR signaling. S1R is an ER–mitochondria interface-associated chaperone that supports inter-organelle communication [67,68] and has been suggested to regulate stress-responsive signaling, including IRE1α-associated pathways [69]. The proposed CBN–S1R/MAM–UPR axis rests on two separate observations rather than on direct evidence: CBN has been reported to preserve mitochondrial function under oxidative stress [31], and S1R modulates ER–mitochondria stress signaling [69]. Direct evidence that CBN binds to or functionally activates S1R in AD-relevant neuronal models is lacking. Thus, the proposed axis remains hypothetical and requires validation using direct CBN–S1R binding or target-engagement assays, S1R knockdown or knockout systems, selective S1R ligands, and AD-relevant neuronal models. Together, the CBDV/CBG-associated UPR marker changes and the hypothesized CBN–S1R/MAM pathway provide testable hypotheses for determining whether minor phytocannabinoids influence adaptive, maladaptive, or neutral UPR responses in AD-relevant systems.

## 6. Multi-Target Rationale and Unresolved Combination Effects

The evidence reviewed here indicates that minor phytocannabinoids are unlikely to act through a single ER stress pathway. Instead, their target profiles extend across several upstream processes that modulate UPR activity and proteostatic burden in AD. CBN is most closely linked to mitochondrial stress pathways and putative ER–mitochondria mechanisms, CBG to oxidative stress-related and inflammatory mechanisms, THCV to inflammatory signaling, and CBC/CBDV to TRP channel-related Ca^2+^ signaling and autophagic proteostasis. These partially overlapping pharmacological profiles may raise questions about potential interactions when these compounds are combined, but pathway complementarity alone does not predict additive or synergistic effects. The entourage concept has been discussed in the broader cannabis literature, particularly in relation to interactions between cannabinoids and terpenes [91,92]. However, these studies do not provide direct evidence for synergistic interactions among the minor phytocannabinoids discussed in this review. Accordingly, the entourage concept should not be used as a mechanistic rationale for prioritizing specific minor-phytocannabinoid combinations. At present, the available evidence is insufficient to identify particular combinations that would be expected to provide superior ER proteostasis modulation. If combination studies are pursued, they should therefore be regarded as exploratory hypothesis-testing experiments using chemically defined compounds, appropriate single-agent controls, defined concentration ratios, concentration–response matrices, and formal interaction analyses to determine whether combined effects are additive, synergistic, neutral, or antagonistic.

Human exposure and tolerability data for related phytocannabinoids, including CBDV in neurodevelopmental conditions and CBD in treatment-resistant epilepsy, support the feasibility of further experimental evaluation, although the safety and clinical applicability of selected minor phytocannabinoid combinations in AD remain unproven [72,93]. In addition, future studies should carefully examine dose optimization, formulation, route of administration, blood–brain barrier penetration, brain exposure, and potential pharmacokinetic interactions between co-administered phytocannabinoids [94].

## 7. Limitations and Future Research Directions

Current evidence supporting the modulation of ER stress by minor phytocannabinoids in AD remains limited, and further validation is needed. First, experimental evidence for the effects of CBG, CBC, CBN, CBDV, and THCV on UPR signaling in AD-relevant neuronal models is sparse. Most proposed mechanisms are based on pharmacological target profiles and indirect evidence from non-neuronal or non-AD systems. In particular, UPR marker changes observed in HTR-8/SVneo trophoblast cells should not be interpreted as evidence of neuroprotection in AD without validation in neuronal systems. Future studies should examine each UPR arm directly after exposure to minor phytocannabinoids. For the PERK branch, relevant markers include phosphorylated PERK, phosphorylated eIF2α, ATF4, and CHOP; for the IRE1α branch, phosphorylated IRE1α and XBP1s should be assessed; and for ATF6 signaling, ATF6 cleavage should be evaluated. These analyses should be performed in patient-derived neurons, human induced pluripotent stem cell-derived cortical organoids, and established AD-relevant cell models [95,96].

Second, in vivo CNS pharmacokinetics of minor phytocannabinoids are incompletely characterized. Blood–brain barrier penetration, brain-to-plasma ratios, metabolic stability, protein binding, and oral bioavailability should be established to determine whether pharmacologically relevant concentrations are achievable in the CNS [94,97]. Given the reported involvement of cannabinoid signaling in ER stress regulation [49], it will also be important to determine whether such CNS exposure is sufficient to modulate the proposed ER stress-related pathways. Although high lipophilicity may facilitate passive membrane permeability, brain exposure can still vary according to molecular properties, formulation, and route of administration [97]. Accordingly, formulation and route of administration need to be optimized, and CNS-targeted delivery strategies may be required to achieve pharmacologically relevant brain concentrations.

An order-of-magnitude comparison further highlights this translational issue. In rats, repeated oral administration at doses up to 100 mg/kg resulted in 24 h brain concentrations of approximately 104 ng/g for CBC, 18.4 ng/g for CBN, and 23.6 ng/g for THCV [94], corresponding approximately to 0.33, 0.06, and 0.08 μM, respectively. These concentrations are below the low-micromolar ranges used in several in vitro studies discussed here, including 1–10 μM for CBDV and CBG and sub- to low-micromolar concentrations associated with CBN activity [31,71]. However, these measurements were obtained 24 h after dosing and do not represent peak brain exposure, and comparable human brain or cerebrospinal fluid (CSF) pharmacokinetic data are lacking. Comparable brain exposure data for CBG and CBDV were also not available from this study. Thus, this comparison should be regarded as a rough feasibility estimate rather than a direct cross-species exposure equivalence. Central proteostatic efficacy should therefore not be inferred from blood–brain barrier permeability or in vitro activity alone but requires evidence of biologically active brain exposure together with in vivo target or pathway engagement. Future studies should determine unbound brain concentrations and relate them directly to compound-specific concentrations required for pathway modulation. Pharmacokinetic studies in rodent AD models and, ultimately, in humans are essential before therapeutic translation.

Beyond exposure feasibility, an additional translational consideration is receptor-mediated CNS safety. CBG and CBN have been reported to interact with CB1R, but the extent to which this activity produces clinically meaningful psychoactive or cognitive effects at pharmacologically relevant exposures remains uncertain. Because CB1R regulates presynaptic neurotransmission and synaptic plasticity [23,27,50], potential behavioral and cognitive liabilities should be evaluated together with CNS exposure, target engagement, and efficacy. This consideration is particularly relevant when developing these compounds for AD, where preservation of cognitive function is a primary therapeutic objective.

Third, a concentration threshold separating adaptive from maladaptive UPR responses has not been defined for any of the five compounds reviewed here, and no study has yet examined protective and injurious concentrations of the same compound within a single AD-relevant neuronal model. The available dose–response data were obtained in different experimental contexts and therefore cannot be assembled into a common preconditioning-to-apoptosis concentration scale. In HTR-8/SVneo trophoblast cells exposed in the absence of an applied stressor, CBDV and CBG were examined at 1–10 μM [71]. Both compounds reduced MTT-based viability at 5 μM after 48 h and at 10 μM after 24 and 48 h, whereas lactate dehydrogenase (LDH) release was detected only at 10 μM. At 5 μM, both compounds increased *HSPA5* and *XBP1s* transcript levels, while CBDV additionally increased *ATF4* transcript levels together with p-eIF2α and CHOP protein levels and initiator caspase-8/-9 activity. However, neither compound activated effector caspases-3/-7 or induced PARP-1 cleavage, indicating that these findings do not define a completed transition to apoptotic cell death [71]. In NSC-34 motor neurons, CBG showed no cytotoxicity across 1–20 μM over 24 h, and 7.5 μM was subsequently used to examine protection against inflammatory conditioned medium [60]. CBN protected HT22 neuronal cells against glutamate- and RSL3-induced oxytosis/ferroptosis with half-maximal effective concentration (EC_50_) values of 1.89 and 0.69 μM, respectively. No apparent CBN cytotoxicity was observed up to 200 μM, whereas cytotoxicity emerged at concentrations of 500 μM or higher [31]. At 5 μM, CBN produced a modest reduction in mitochondrial membrane potential and increased Nrf2, heme oxygenase-1 (HO-1), superoxide dismutase 2 (SOD2), and glutathione peroxidase 4 (GPX4) expression, while ATF4 was not significantly altered [31]. These findings indicate a protective mitochondrial response at this concentration but do not establish adaptive UPR preconditioning. Because these studies differ in cell type, species, exposure duration, presence or absence of an applied stressor, and the readouts used, their concentrations are not directly comparable, and comparable dose-transition data are unavailable for CBC and THCV. Defining compound- and cell type-specific therapeutic windows will therefore require concentration–response studies performed within the same AD-relevant neuronal model, in which adaptive-associated UPR markers, such as GRP78/BiP and XBP1s, are evaluated in parallel with maladaptive/apoptotic-associated readouts, including CHOP, caspase activation, and cell death [12,90].

Fourth, the hypothesized CBN–S1R/MAM–UPR mechanism and CBDV/CBG-associated UPR marker changes require validation in AD-relevant neuronal systems. Future studies should combine direct assessment of UPR markers, neuronal AD models, defined exposure conditions, and S1R perturbation approaches to determine whether these responses are adaptive, maladaptive, or neutral.

Finally, clinical translation requires appropriate ER stress biomarkers for patient stratification and pharmacodynamic monitoring. Given the neuropathological evidence of UPR activation in AD and the limited availability of clinically validated ER stress-related biomarkers, future studies may explore CSF- or blood-derived indicators of PERK/eIF2α signaling and XBP1s-related responses as tentative pharmacodynamic endpoints. However, these markers should not be regarded as established AD biomarkers [9,34]. Future clinical studies may be best positioned in mild cognitive impairment or very early symptomatic AD, when residual proteostatic capacity may still be sufficient to evaluate whether minor phytocannabinoids can modulate ER stress-related pharmacodynamic endpoints.

## 8. Conclusions

Chronic ER stress and UPR dysregulation intersect with Aβ deposition, abnormal tau phosphorylation, neuroinflammation, mitochondrial impairment, defective autophagy, and progressive proteostasis failure. Therefore, minor phytocannabinoids may serve as pharmacological probes for testing whether the upstream modulation of these processes can support ER proteostasis in AD-relevant systems. Direct validation in patient-derived neural models and organoids, in vivo studies, pharmacokinetic analyses, and early translational biomarker studies is required before therapeutic claims can be made.

## Figures and Tables

**Figure 1:**
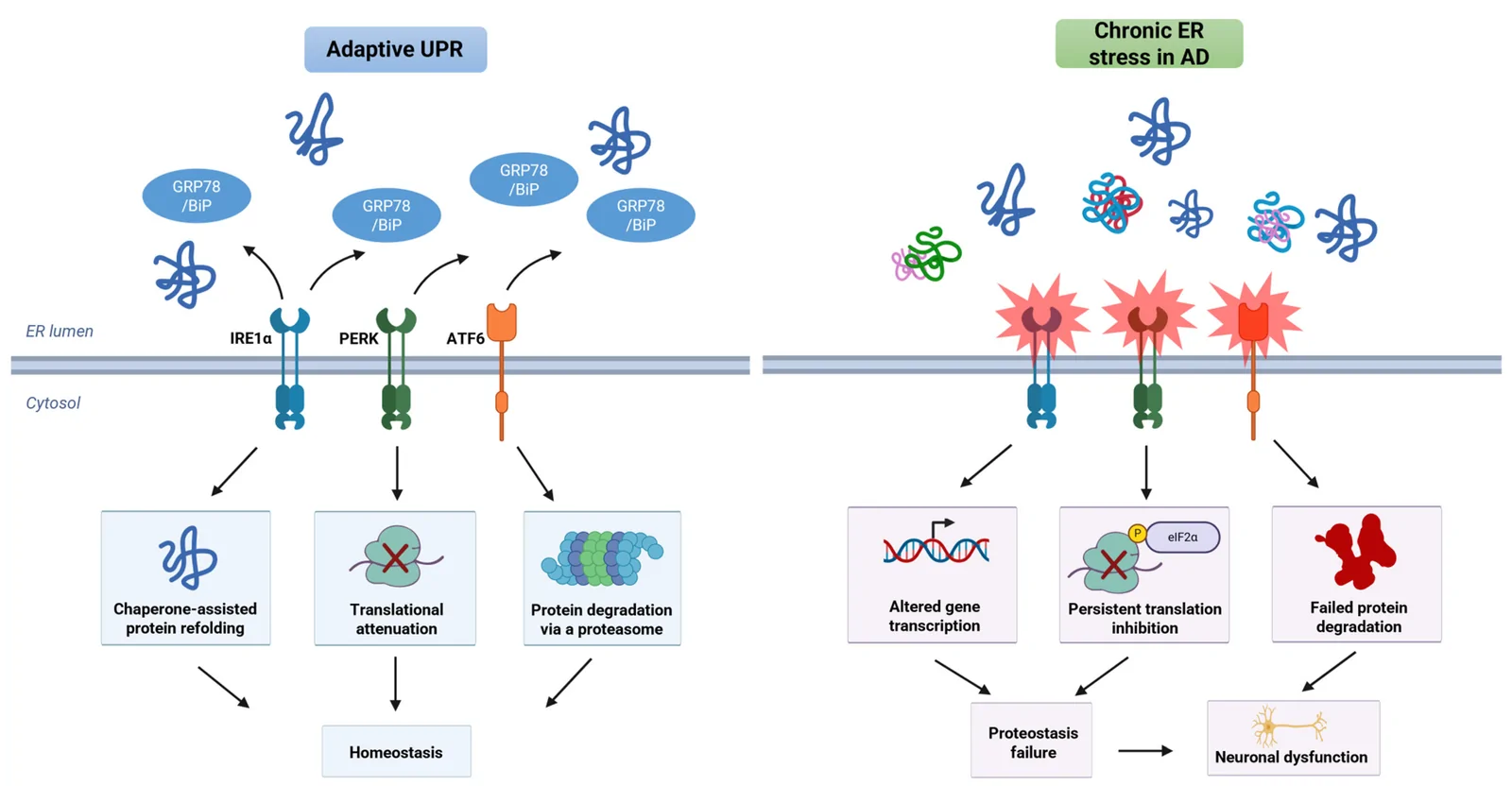
Adaptive versus maladaptive UPR signaling in Alzheimer’s disease (AD). Under acute or reversible endoplasmic reticulum (ER) stress, an adaptive unfolded protein response (UPR) is engaged, driving chaperone-assisted refolding, translational attenuation, and protein degradation to preserve proteostasis and neuronal homeostasis. In AD, persistent ER stress is associated with maladaptive UPR signaling, characterized by altered gene transcription, persistent translational inhibition, and failed protein degradation, ultimately contributing to proteostasis failure and neuronal dysfunction. Arrows indicate the direction of UPR signaling and its downstream effects on proteostasis and neuronal function. Created in BioRender. Lee, H. (2026): https://BioRender.com/8mymex8.

**Figure 2:**
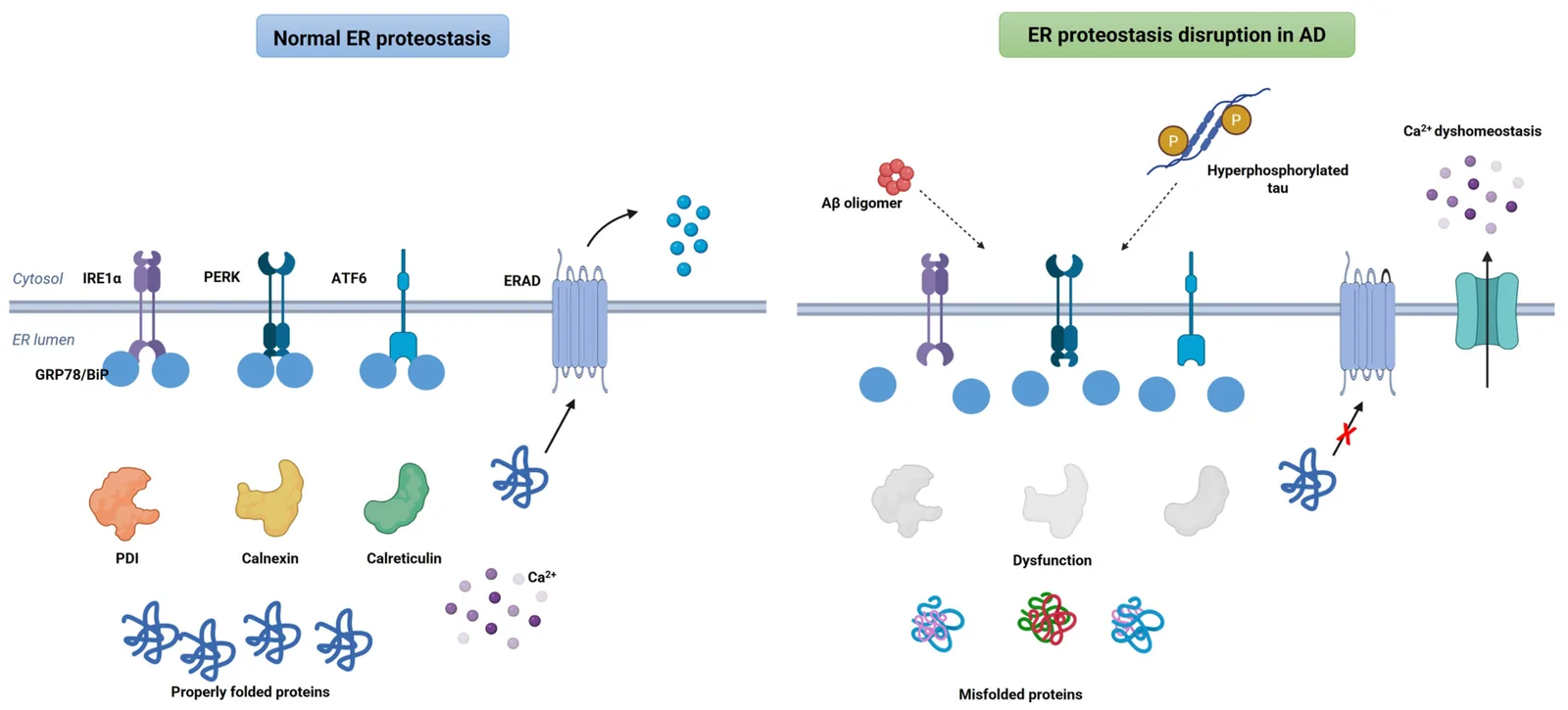
Disruption of ER proteostasis in Alzheimer’s disease (AD). In healthy neurons, ER protein homeostasis depends on coordinated folding, Ca^2+^ regulation, and ER-associated degradation (ERAD) and is supported by luminal chaperones and foldases such as GRP78/BiP, PDI, calnexin, and calreticulin. In AD, amyloid-β (Aβ) oligomers and hyperphosphorylated tau are associated with the disruption of ER proteostasis, accompanied by altered UPR sensor signaling through IRE1α, PERK, and ATF6; dysregulation of GRP78/BiP and ER folding machinery; Ca^2+^ dyshomeostasis; accumulation of misfolded proteins; and defective ERAD. These changes may increase the ER proteotoxic burden and chronic ER stress, thereby contributing to neuronal vulnerability. Solid arrows indicate the direction of ERAD-associated protein trafficking and Ca^2+^ release from the ER; dotted arrows indicate the proposed effects of Aβ oligomers and hyperphosphorylated tau on ER proteostasis; the red cross indicates impaired ERAD. Created in BioRender. Lee, H. (2026): https://BioRender.com/8mymex8.

**Figure 3:**
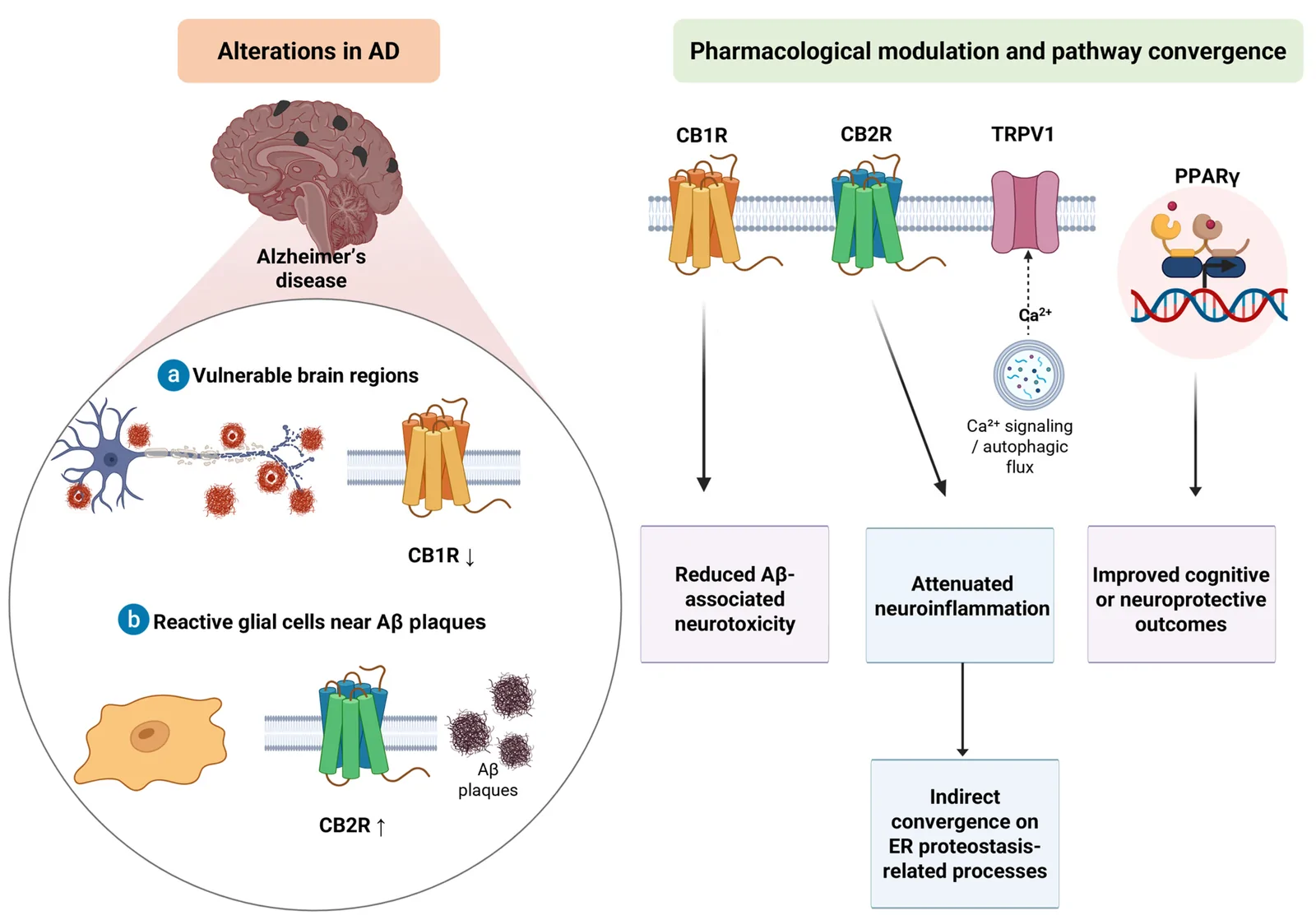
Mechanistic summary of hypothetical pathway convergence between endocannabinoid system (ECS) alterations and ER proteostasis in Alzheimer’s disease (AD). In Alzheimer’s disease (AD), neuronal CB1R expression is reduced in AD-vulnerable areas such as the hippocampus and cortex, whereas CB2R expression is increased in reactive glial cells surrounding amyloid-β (Aβ) plaques. Pharmacological modulation of ECS-related and phytocannabinoid-sensitive targets, including CB1R, CB2R, TRPV1, and PPARγ, has been linked to reduced Aβ-associated neurotoxicity, attenuated neuroinflammation, regulation of Ca^2+^ signaling and autophagic flux, and improved cognitive or neuroprotective outcomes in experimental models. These pathway-level effects may indirectly converge on ER proteostasis-related processes. The schematic is intended as a mechanistic summary, not as evidence of therapeutic efficacy in AD. Solid arrows indicate the proposed direction of pathway modulation and its potential downstream effects; the dotted arrow indicates TRPV1-associated Ca^2+^ signaling and autophagic flux. Downward and upward arrows next to CB1R and CB2R indicate decreased and increased expression in AD, respectively. Created in BioRender. Lee, H. (2026): https://BioRender.com/8mymex8.

**Figure 4:**
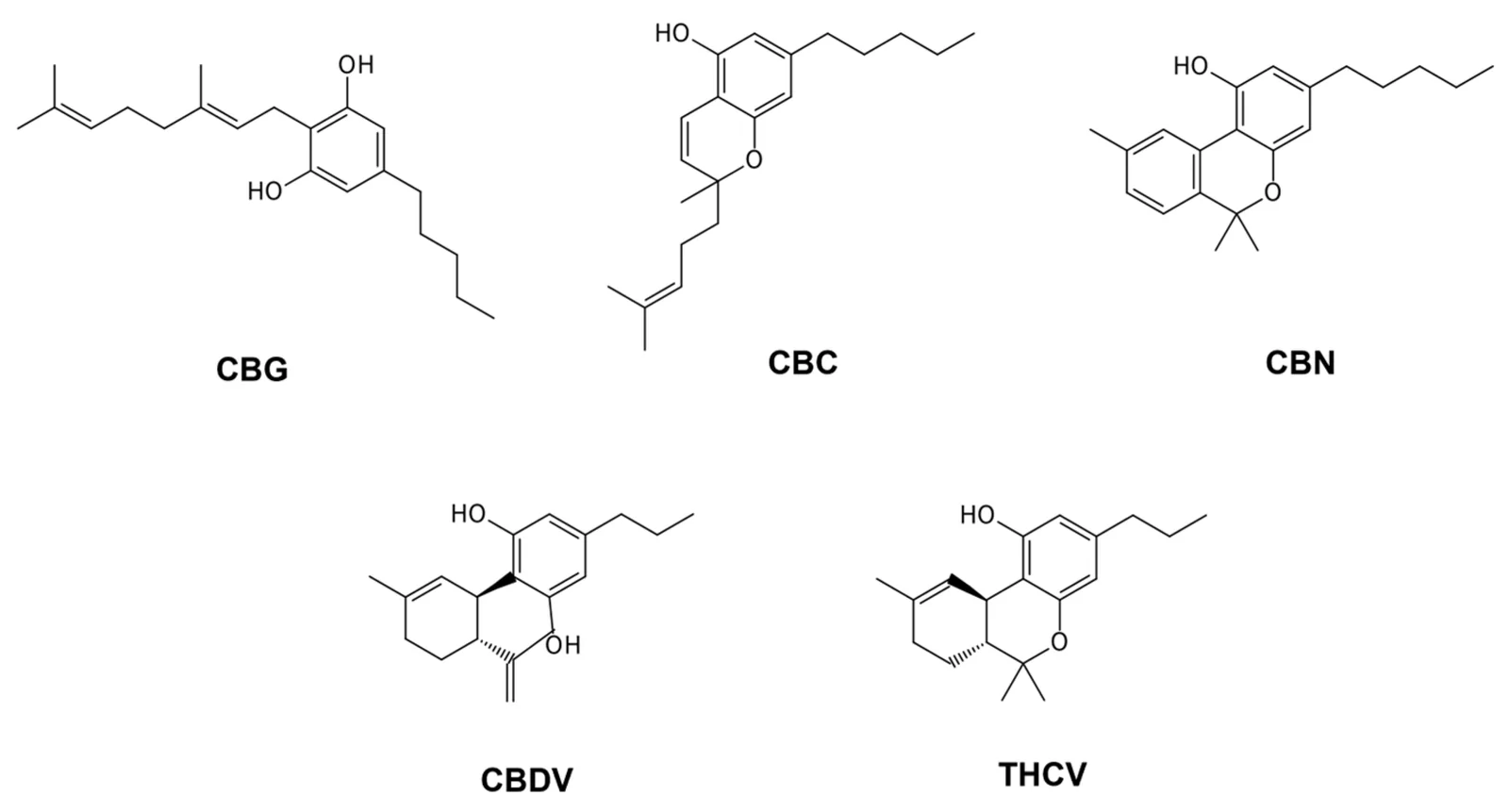
Chemical structures of the minor phytocannabinoids discussed in this review. The chemical structures of cannabigerol (CBG), cannabichromene (CBC), cannabinol (CBN), cannabidivarin (CBDV), and tetrahydrocannabivarin (THCV) are shown to facilitate comparison of their structural features. Created in BioRender. Lee, H. (2026): https://BioRender.com/8mymex8.

**Figure 5:**
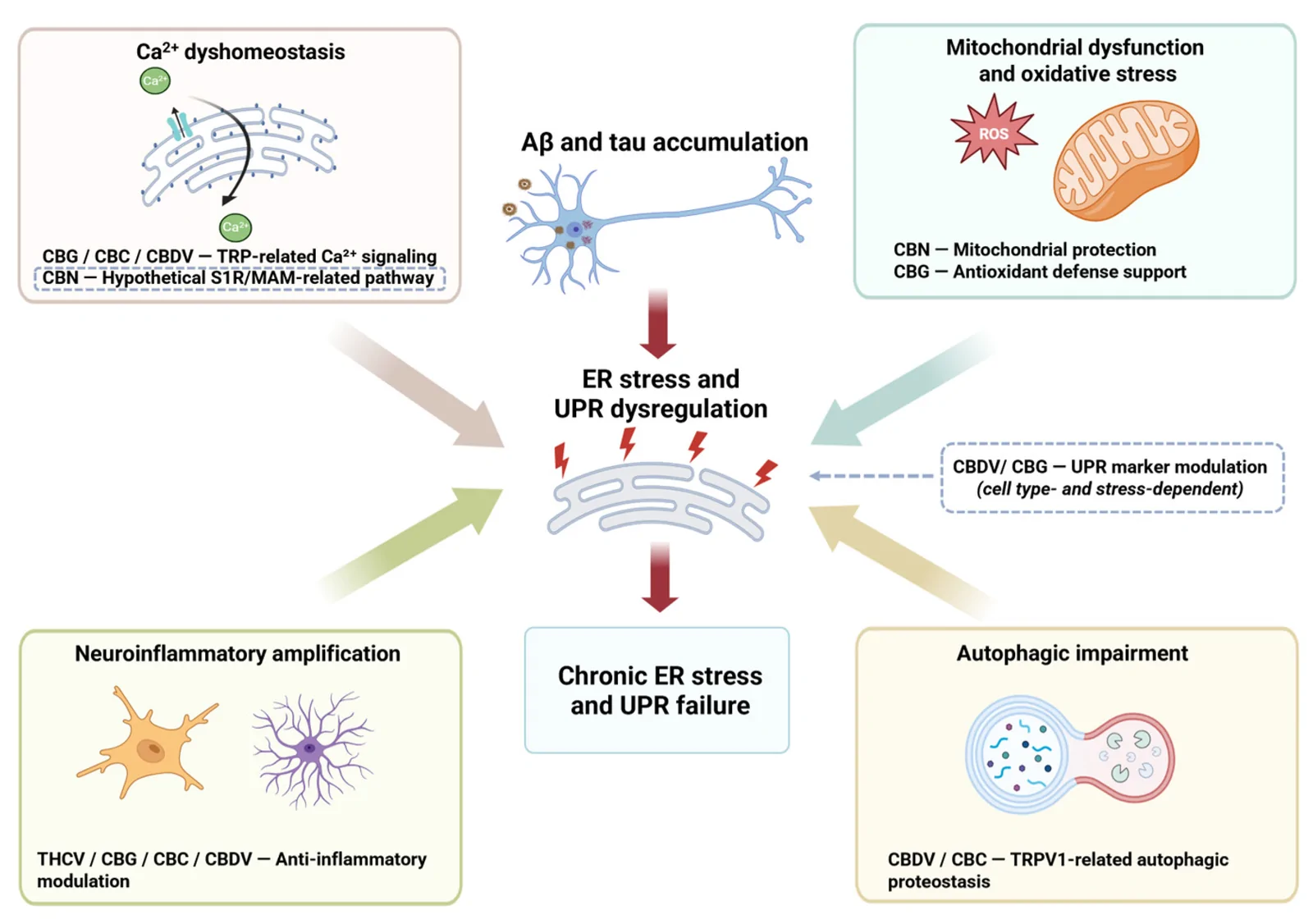
Mechanistic summary of a hypothetical model of action for minor phytocannabinoid modulation of ER stress-linked pathological processes in Alzheimer’s disease (AD). The schematic depicts amyloid-β (Aβ) and tau accumulation, Ca^2+^ dyshomeostasis, mitochondrial injury with redox imbalance, neuroinflammatory amplification, and defective autophagy as pathological processes that may intensify endoplasmic reticulum (ER) stress and disrupt unfolded protein response (UPR)-dependent proteostasis in AD. Representative minor phytocannabinoids are placed according to their proposed relevance to TRP-related Ca^2+^ signaling, hypothesized S1R/MAM-related signaling, mitochondrial and oxidative stress-related regulation, anti-inflammatory modulation, and TRPV1-related autophagic flux. CBDV and CBG are additionally shown in relation to cell type- and stress-dependent UPR marker modulation. This schematic presents a hypothesis-based model rather than evidence of established therapeutic efficacy or validated mechanisms in AD. Solid arrows indicate the proposed contribution of pathological processes to ER stress and UPR dysregulation and their downstream consequences. The dashed arrow and dashed boxes indicate hypothesized or context-dependent relationships, including the link between CBDV/CBG-associated UPR marker changes and ER stress, that remain to be validated experimentally. CBG, cannabigerol; CBC, cannabichromene; CBN, cannabinol; CBDV, cannabidivarin; THCV, tetrahydrocannabivarin; TRP, transient receptor potential; S1R, sigma-1 receptor; MAM, mitochondria-associated membrane; TRPV1, transient receptor potential vanilloid 1. Created in BioRender. Lee, H. (2026): https://BioRender.com/8mymex8.

**Table 1:** Pharmacological profiles of representative minor phytocannabinoids and their proposed relevance to ER stress modulation in Alzheimer’s disease.

Compound	Reported Targets/ Pathways	Key Properties	ER Stress-Relevant Mechanisms	References
CBG	CB1R/CB2R partial agonist PPARγ TRPM8	Anti-inflammatory Antioxidant defense support Restoration of catalase, SOD, and GSH parameters Ca^2+^ modulation	Attenuation of inflammatory and oxidative stress-associated contributors to ER stress TRPM8-related Ca^2+^ modulation and PPARγ-related anti-inflammatory signaling	[57,59,60,61,62,63]
CBC	TRPA1 Low-efficacy TRPV1 activity Weak CB1R/CB2R activity	Anti-inflammatory Regulation of Ca^2+^ influx Potential TRPV1-mediated autophagy	TRPA1/TRPV1-related Ca^2+ ^signaling Reduction in inflammatory mediators Context-dependent TRPV1-linked autophagic proteostasis	[26,51,57,64,65]
CBN	CB1R/CB2R partial agonist Mitochondrial targets Hypothetical S1R/MAM-related pathway	Mitochondrial protection Reduction in oxidative cell death Anti-apoptotic effects	Potential attenuation of mitochondrial ROS-driven ER stress Potential preservation of ER–mitochondria homeostasis Hypothesized S1R/MAM-related ER–mitochondria signaling	[31,66,67,68,69,70]
CBDV	TRPV1 Low-affinity CB1R/CB2R activity	Anti-inflammatory Anticonvulsant	Cell type- and stress-dependent PERK/IRE1α pathway marker modulation TRPV1-related Ca^2+^ signaling Context-dependent TRPV1-related autophagic regulation	[45,58,71,72,73,74,75]
THCV	CB1R/CB2R modulation PPARγ-related pathways	Anti-inflammatory Metabolic regulation Reduction in inflammatory activation	CB2R- and PPARγ-related anti-inflammatory signaling Potential indirect attenuation of inflammatory UPR amplification	[30,76,77,78]

Abbreviations: AD, Alzheimer’s disease; CB1R/CB2R, cannabinoid receptors 1 and 2; CBG, cannabigerol; CBC, cannabichromene; CBN, cannabinol; CBDV, cannabidivarin; ER, endoplasmic reticulum; GSH, glutathione; IRE1α, inositol-requiring enzyme 1α; MAM, mitochondria-associated membrane; PERK, protein kinase R-like ER kinase; PPARγ, peroxisome proliferator-activated receptor γ; ROS, reactive oxygen species; S1R, sigma-1 receptor; SOD, superoxide dismutase; THCV, tetrahydrocannabivarin; TRPA1, transient receptor potential ankyrin 1; TRPM8, transient receptor potential melastatin 8; TRPV1, transient receptor potential vanilloid 1; UPR, unfolded protein response. Note: The proposed mechanisms are based on available pharmacological and preclinical evidence and should not be regarded as established therapeutic mechanisms in AD. Target or pathway overlap with ER stress-relevant processes should be regarded as hypothesis-generating and does not establish direct ER stress modulation or therapeutic benefit in AD. The proposed CBN–S1R/MAM pathway is hypothetical, and direct CBN binding to or functional engagement of S1R has not been demonstrated.

## Data Availability

No new data were created or analyzed in this study. Data sharing is not applicable to this article.

## Abbreviations

The following abbreviations are used in this manuscript:

Aβ	Amyloid-β
AD	Alzheimer’s disease
ATF4	Activating transcription factor 4
ATF6	Activating transcription factor 6
CB1R	Cannabinoid receptor 1
CB2R	Cannabinoid receptor 2
CBC	Cannabichromene
CBD	Cannabidiol
CBDV	Cannabidivarin
CBG	Cannabigerol
CBN	Cannabinol
CHOP	C/EBP homologous protein
CNS	Central nervous system
CSF	Cerebrospinal fluid
ECS	Endocannabinoid system
eIF2α	Eukaryotic initiation factor 2α
ER	Endoplasmic reticulum
ERAD	ER-associated degradation
GSH	Glutathione
GRP78/BiP	Glucose-regulated protein 78/binding immunoglobulin protein
GRP94	Glucose-regulated protein 94
HSPA5	Heat shock protein family A member 5
IP_3_R	Inositol 1,4,5-trisphosphate receptor
IRE1α	Inositol-requiring enzyme 1α
JNK	c-Jun N-terminal kinase
MAM	Mitochondria-associated membrane
NF-κB	Nuclear factor-kappa B
Nrf2	Nuclear factor erythroid 2-related factor 2
PARP-1	Poly(ADP-ribose) polymerase-1
PDI	Protein disulfide isomerase
PERK	Protein kinase R-like ER kinase
PPARγ	Peroxisome proliferator-activated receptor γ
p-eIF2α	Phosphorylated eIF2α
ROS	Reactive oxygen species
RyR	Ryanodine receptor
S1R	Sigma-1 receptor
SERCA	Sarco/endoplasmic reticulum Ca^2+^-ATPase
SNO	S-nitrosylation
SOD	Superoxide dismutase
THC	Δ^9^-tetrahydrocannabinol
THCV	Tetrahydrocannabivarin
TRP	Transient receptor potential
TRPA1	Transient receptor potential ankyrin 1
TRPM8	Transient receptor potential melastatin 8
TRPV1	Transient receptor potential vanilloid 1
UPR	Unfolded protein response
XBP1	X-box binding protein 1
XBP1s	Spliced X-box binding protein 1
